# Combinatorial treatment using targeted MEK and SRC inhibitors synergistically abrogates tumor cell growth and induces mesenchymal-epithelial transition in non-small-cell lung carcinoma

**DOI:** 10.18632/oncotarget.5031

**Published:** 2015-08-17

**Authors:** Kian Ngiap Chua, Li Ren Kong, Wen Jing Sim, Hsien Chun Ng, Weijie Richard Ong, Jean Paul Thiery, Hung Huynh, Boon Cher Goh

**Affiliations:** ^1^ Cancer Science Institute of Singapore, National University of Singapore, Singapore; ^2^ Institute of Molecular and Cell Biology, A*STAR, Singapore; ^3^ National Cancer Centre, Singapore; ^4^ Department of Biochemistry, Yong Loo Lin School of Medicine, National University of Singapore, Singapore; ^5^ Department of Hematology-Oncology, National University Hospital, Singapore; ^6^ National University Cancer Institute, Singapore

**Keywords:** mesenchymal-epithelial transition inducer, MEK and SRC inhibitor combination treatment, PD0325901, saracatinib, non-small-cell lung cancer

## Abstract

Oncogenesis in non-small cell lung cancer (NSCLC) is regulated by a complex signal transduction network. Single-agent targeted therapy fails frequently due to treatment insensitivity and acquired resistance. In this study, we demonstrate that co-inhibition of the MAPK and SRC pathways using a PD0325901 and Saracatinib kinase inhibitor combination can abrogate tumor growth in NSCLC. PD0325901/Saracatinib at 0.25:1 combination was screened against a panel of 28 NSCLC cell lines and 68% of cell lines were found to be sensitive (IC_50_ < 2 μM) to this combination. In Snail1 positive NSCLC lines, the drug combination complementarily enhanced mesenchymal-epithelial transition (MET), increasing both E-cadherin and Plakoglobin expression, and reducing Snail1, FAK and PXN expression. In addition, the drug combination abrogated cell migration and matrigel invasion. The co-inhibition of MAPK and SRC induced strong G1/G0 cell cycle arrest in the NSCLC lines, inhibited anchorage independent growth and delayed tumor growth in H460 and H358 mouse xenografts. These data provide rationale for further investigating the combination of MAPK and SRC pathway inhibitors in advanced stage NSCLC.

## INTRODUCTION

Lung cancer is one of the leading causes of cancer mortality in many countries [1–3]. Approximately 85% of all lung cancers are non-small cell lung cancers (NSCLC), which can be further subclassified into squamous cell carcinoma, large cell carcinoma and adenocarcinoma. Despite significant therapeutic advances made in recent decades, the 5-year survival rate for lung cancer remains at less than 17% [2, 3]. In general, the effectiveness of chemotherapy, radiotherapy and surgery have been unsatisfactory, especially in the treatment of advanced NSCLC [4]. Thus, new treatment strategies that target the molecular and cellular events underlying the development of this fatal disease are urgently needed.

The introduction of targeted therapeutics for cancer treatment in recent years has significantly changed the practice of medical oncology and many targeted therapeutics are being validated in various stages of clinical development. Some of these targeted compounds, such as Gefitinib and Erlotinib, have been approved for first-line treatment to treat advanced NSCLC harboring sensitive epidermal growth factor receptor (EGFR) mutations [5, 6]. Unfortunately, these targeted therapies will eventually limit the effectiveness of traditional chemotherapies in the metastatic disease due to the relatively rapid acquisition of drug resistance developed by the tumor cells [7]. In NSCLC, studies have shown that secondary mutations (T790M) in the EGFR kinase domain can promote resistance to EGFR inhibitor treatment [8]. Furthermore, EGFR can crosstalk with other growth factor receptors, such as insulin-like growth factor-I receptor (IGF-IR), and reduces the effectiveness of Erlotinib treatment through the activation of the AKT signaling pathway [9].

Studies have therefore begun to investigate on the application of targeted drug combinations to abrogate signaling pathways cross-talk and restore treatment sensitivity [10, 11]. For example, Sos et al demonstrated in NSCLC cell lines that combined blockade of phosphatidylinositol-4,5-bisphosphate 3-kinase (PI3K) and mitogen-activated protein kinase (MAPK) pathways could overcome the reciprocal pathway activation induced by inhibitor-mediated feedback loops and resulted in significant increase in apoptosis and tumor shrinkage [10]. Likewise, Legrier et al also showed that combined inhibition of MAPK and mammalian target of rapamycin (mTOR) synergistically suppressed proliferation in NSCLC cell lines and induced regression of xenograft tumors [11]. Due to the shear diversity of this topic, it is foreseeable that other targeted drug combinations may be proven to be effective and may become viable lung cancer treatments in the future.

Studies have also focused on non-genetic mechanisms such as Epithelial Mesenchymal Transition (EMT) as a cause of tumor cell dissemination and drug resistance in cancer therapy [4, 12–15]. EMT is a crucial mechanism for carcinoma progression, as it provides routes for *in situ* carcinoma cells to dissociate and become motile, leading to localized invasion and metastatic spread. Indeed, bone, brain, lymph nodes, liver and adrenal glands metastases are a very common secondary localization of disease in lung cancer patients, with 30–40% of patients developing brain and bone metastases in the course of their disease [16, 17]. Targeting EMT therefore represents an important therapeutic strategy for the treatment of advanced NSCLC exhibiting highly invasive and metastatic phenotype [14, 15]. We have hypothesized that some targeted therapeutics, whilst initially optimized as anti-proliferative agents, may also inhibit EMT initiation and sustenance, since they are both regulated by similar signaling pathways that these compounds were designed to inhibit [15]. However, in-depth investigations to characterize existing targeted drugs on EMT modulating properties are still limited to date. We had recently discovered through a novel cell-based, high-content EMT screening assay, that two targeted compounds, PD0325901 and Saracatinib, selective inhibitors of MEK and SRC kinases respectively, were also potent EMT modulators that could interfere with EGF, HGF, and IGF-1 induced EMT signaling in a NBT-II EMT reporter cell line [14].

In this study, we investigate whether PD0325901 and Saracatinib co-treatment can synergistically suppress cell proliferation and tumorigenicity in NSCLC lines. We also evaluate the impact of PD0325901 and Saracatinib in modulating the EMT process via induction of Mesenchymal-Epithelial Transition (MET) in NSCLC lines. Specifically, we also determine whether PD0325901 and Saracatinib in combination can induce strong antitumor and MET response across multiple NSCLC lines.

## RESULTS

### Cell proliferation inhibition effects of PD0325901 or Saracatinib single drug treatments on lung cancer cell lines

**Figure 1:**
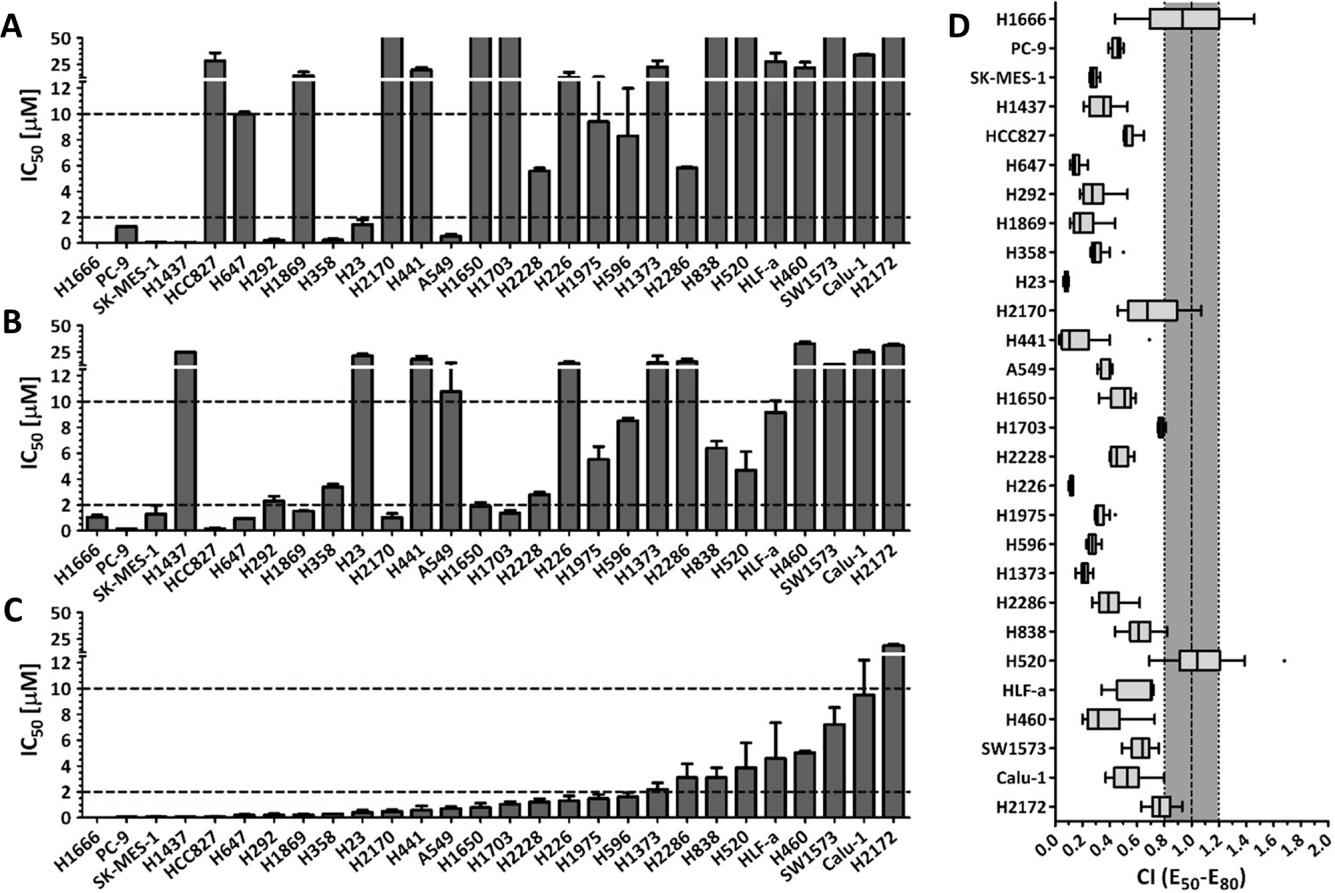
The combination of MEK inhibitor PD0325901 with SRC inhibitor Saracatinib promoted synergistic inhibition of cell growth in NSCLC cell lines Cell proliferation IC_50_ plots (mean ± SD) for a panel of 28 NSCLC cell lines treated with PD0325901 **A.** Saracatinib **B.** or at a fixed PD0325901 to Saracatinib ratio of 0.25:1 **C.** for 72 h. Data were tabulated from three independent experiment sets. IC_50_ < 2 μM indicates cell lines are sensitive to drug (lower dotted line), IC_50_ > 10 μM indicates cell lines are insensitive to drug (upper dotted line). **D.** combination index (CI) box plots of PD0325901 and Saracatinib co-treatment at the ratio of 0.25:1 on the cell line panel. Combination index of CI < 0.8 indicates synergism, CI from 0.8 to 1.2 indicates additive effect, and CI > 1.2 indicates antagonism.

We investigated on the proliferation inhibition effects of PD0325901 and Saracatinib as single drug therapies on a collection of 28 lung cancer cell lines. We found that only 8 out of 28 cell lines (29%) were sensitive to PD0325901 treatment (cell proliferation IC_50_ < 2 μM), while 15 cell lines (54%) were considered resistant to this compound (cell proliferation IC_50_ > 10 μM) (Fig. 1A). In general, the growth inhibition response to PD0325901 varied widely, with cell lines responding highly sensitively (H1437 and H1666, IC_50_ < 50 nM), to cell lines that were highly resistant (H1650 and H2170, IC_50_ > 100 μM). For Saracatinib single drug treatment, 9 cells lines (32%) were observed to be sensitive, while 11 cell lines (39%) were found to be resistant (Fig. 1B). The growth inhibition response to Saracatinib was observed to be less varied, with the IC_50_ ranging from 150 nM (PC-9) to 33 μM (H460). No correlation between the cell lines’ sensitivity to these two compounds was observed.

### PD0325901 synergized with Saracatinib co-treatment to reduce cell proliferation in lung cancer cell lines

We next investigated on the proliferation inhibition effects of PD0325901 (PD) and Saracatinib (AZ) co-treatment on the lung cancer cell lines. We generated the drug response profiles of three different PD0325901 / Saracatinib co-treatments at fixed PD:AZ combination ratios of 4:1, 1:1 and 0.25:1 for each cell line (Fig. 1C and Supplemental Fig. 1). We then performed synergism analysis by comparing the growth inhibition effects of the single drugs to the drug combinations. The drug combination indices for between the 50%–80% growth inhibition range were then calculated using the Loewe Additivity model that had been used extensively in drug combination studies [10, 11, 14, 18]. We observed that PD:AZ at 0.25:1 ratio was the optimal drug ratio for most of the tested cell lines, as the combination ratio tend to generate more synergistic CI values (Supplemental Fig. 2). We observed that when PD:AZ at 0.25:1 combination was screened against the cell lines, 19 cell lines (68%) were now found to be sensitive, and only 1 cell line (4%) remained resistant to this combination (Fig. 1C). We also observed that the drug combination performed synergistically on 26 cell lines (CI < 0.8), with strong synergism observed on 17 cell lines (CI < 0.5) (Fig. 1D).

### PD0325901 and Saracatinib induced Mesenchymal-Epithelial transition in Snail1 positive NSCLC lines

To further our investigation on whether PD0325901, Saracatinib and their combination treatment could induce MET, we selected 6 cells lines (Calu-1, H23, H358, H460, H838 and H1373) which exhibited positive basal EMT transcription factor Snail1 expression. In addition, PD0325901 and Saracatinib combination treatment had presented good synergism on these cell lines (Fig. 1D and Supplemental Fig. 2). Subsequent *in vitro* combination studies on these cell lines will be tested at the PD:AZ optimal drug ratio of 0.25:1, as observed previously.

**Figure 2:**
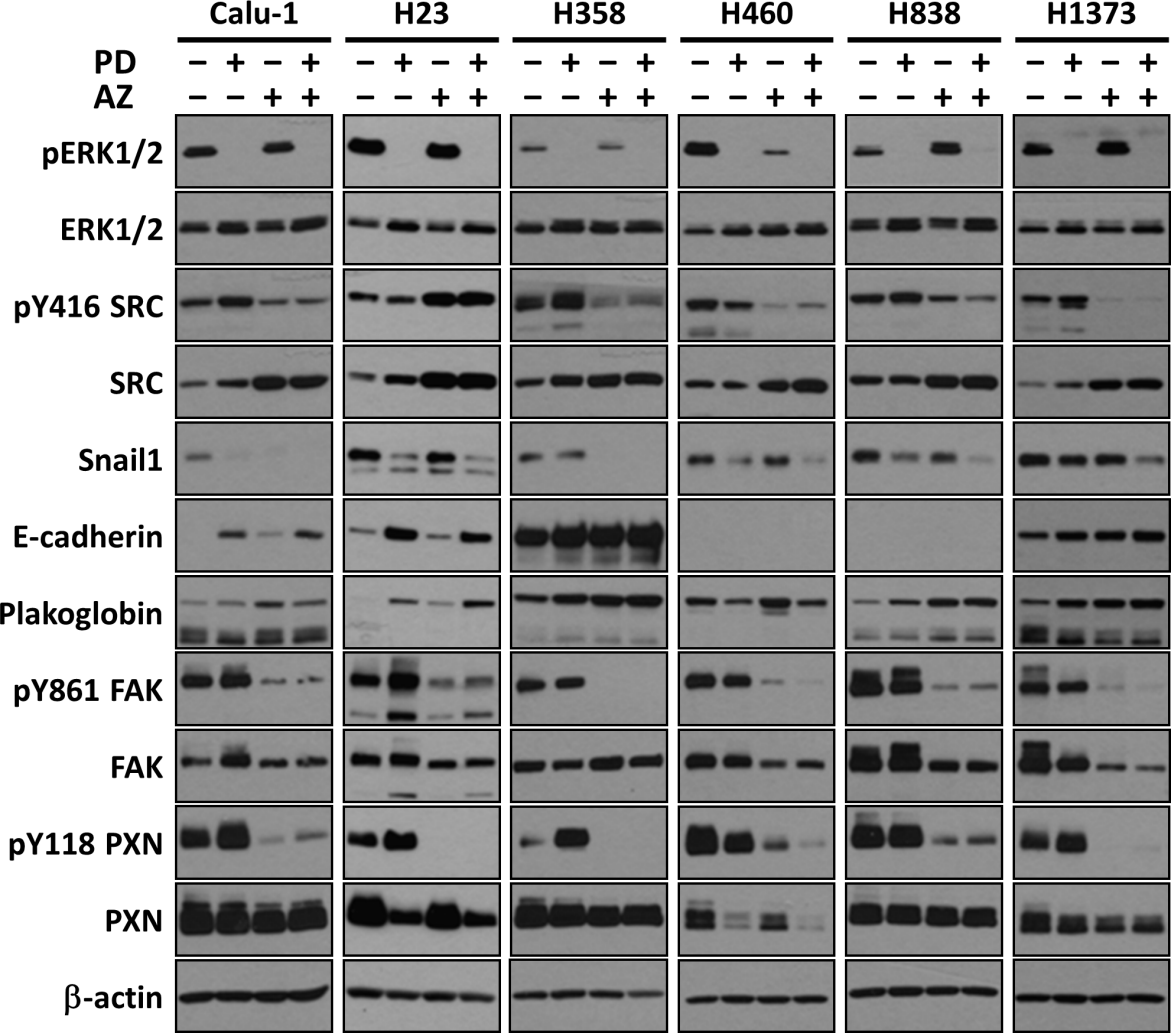
PD0325901, Saracatinib and the combination treatment induced MET in Snail1 positive NSCLC lines Representative Westernblot images showing the effects of PD (1 μM), AZ (4 μM) and their combination treatment on inhibiting the direct targets ERK and SRC, and modulating EMT-related protein markers (E-cadherin and Plakoglobin epithelial markers; and Snail1, FAK and PXN mesenchymal markers). β-actin shown as loading control.

We first demonstrated that MEK inhibition by PD0325901 at 1 μM can potently nullify the phosphorylation of its immediate downstream target ERK1/2 in these cell lines (Fig. 2). Likewise, we showed that SRC inhibition by Saracatinib at 4 μM was able to down-regulate phosphorylation of SRC at Y416 and its immediate downstream targets FAK at Y861 and PXN at Y118. We also validated the target selectivity of these two compounds whereby individually, they could not down-regulate the phosphorylation of each other's target signaling proteins. These results highlighted the potential advantage of using highly selective compounds in combinations, whereby modulation of specific targets can be customized with different drug combination.

We next showed that MET can be effected by both PD0325901 and Saracatinib in NSCLC cell lines (Fig. 2). We observed that different cell lines exhibit different degrees of MET response against the two compounds individually. For example, PD0325901 induced a stronger down-regulation of Snail1 in H838 as compared to H358 line, while Saracatinib induced a stronger Snail1 down-regulation in H358 compared to H838. Similar observations were also found when comparing the individual drug induced E-cadherin changes between Calu-1 and H1373 lines. Overall, we consistently observed a down-regulation of Snail1 expression, in concordance with the up-regulation of both E-cadherin and Plakoglobin epithelial markers in the drug treated samples compared to untreated control, indicative of MET induction [19]. Interestingly, we observed that the drug combination together can complementarily enhance MET induction, increasing both E-cadherin and Plakoglobin expression, and further reducing Snail1, FAK and PXN expression in the cell lines, compared to the single drug treated conditions.

### PD0325901 and Saracatinib increased E-cadherin and Plakoglobin expression and localization at the cell-cell contacts

**Figure 3:**
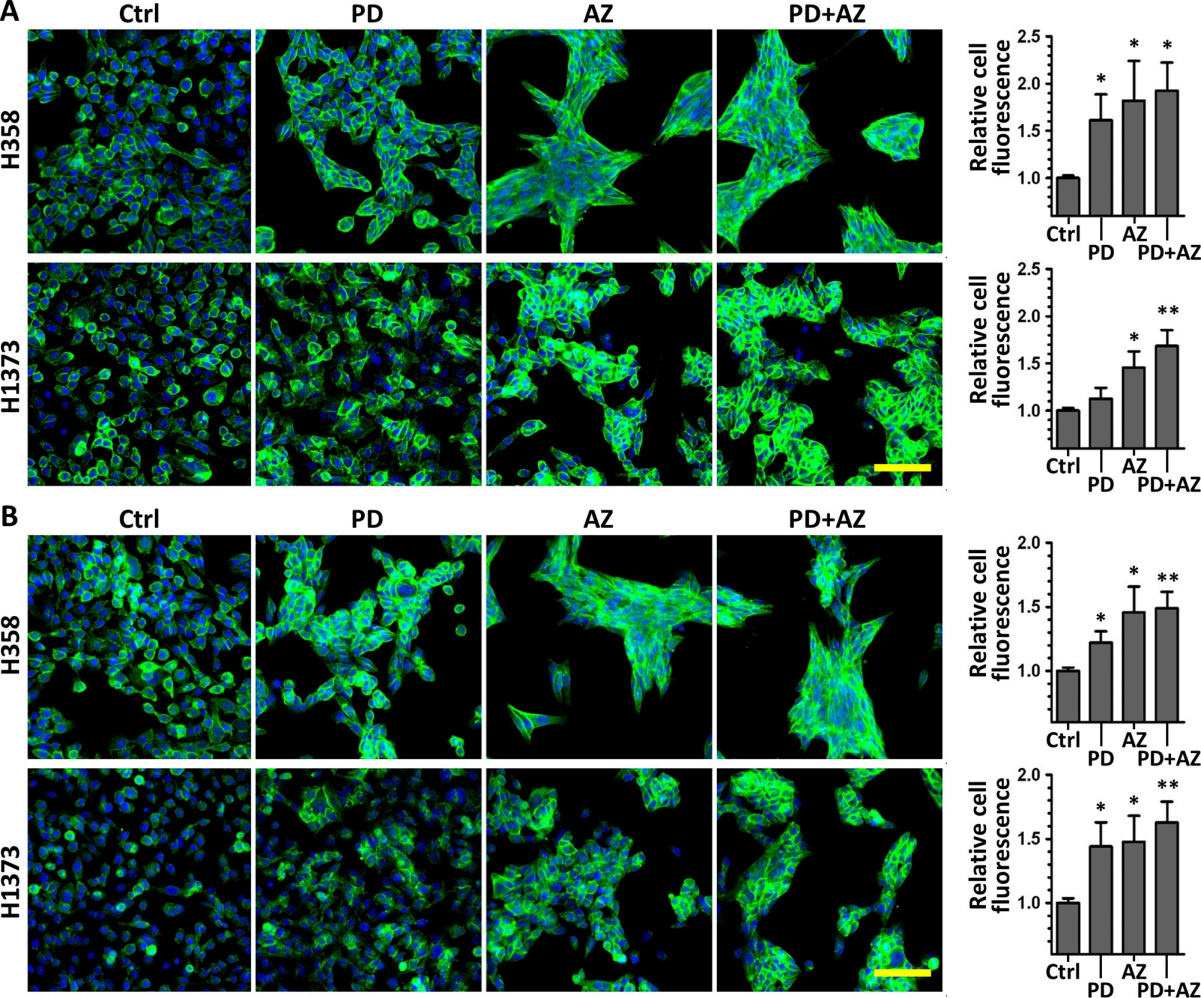
PD0325901, Saracatinib and the combination treatment increased epithelial markers E-cadherin and Plakoglobin expression and localization at the cell-cell contacts H358 and H1373 were treated with DMSO control (Ctrl), or with PD (1 μM), AZ (4 μM) or their combination for 48 h to induce MET. E-cadherin or Plakoglobin immunofluorescence imaging was performed and the cell fluorescence for each marker was quantified. **A.** representative images of E-cadherin stained cells (green) under various drug treated conditions. **B.** representative images of Plakoglobin stained cells (green) under various drug treated conditions. Cell nuclei were counterstained with Hoechst 33342 (blue). Scale bar: 100 μm. Graphs show the relative cell fluorescence intensity (mean ± SD) of each treated group compared to the control group, calculated from at least three independent experiments. *,*P* < 0.05; **,*P* < 0.01 compared with Ctrl group.

We conducted immunofluorescence staining experiments to further analyze the MET induction response through quantifying and comparing the functional expression and localization of E-cadherin (Fig. 3A) and Plakoglobin (Fig. 3B) in untreated and drug treated H358 and H1373 cell lines. Morphologically, we immediately observed that the drug treated cells exhibited increased cell compactness compared with untreated cells. Consistent with the findings above, we observed that both PD0325901 and Saracatinib single treatments could increase E-cadherin and Plakoglobin expression, with drug combination treatment showing the most significant increase in expression levels (E-cadherin 1.92 ± 0.30 fold and Plakoglobin 1.49 ± 0.13 fold in H358 line; E-cadherin 1.68 ± 0.17 fold and Plakoglobin 1.62 ± 0.17 fold in H1373 line), compared to untreated controls. We also observed that both PD0325901 and Saracatinib single treatments and the drug combination treatment to an even greater extent could induce a higher degree of localization of E-cadherin and Plakoglobin at the cell-cell contacts, as compared to controls.

### MET induction by PD0325901 and Saracatinib reduced cell migratory and invasive behavior

**Figure 4:**
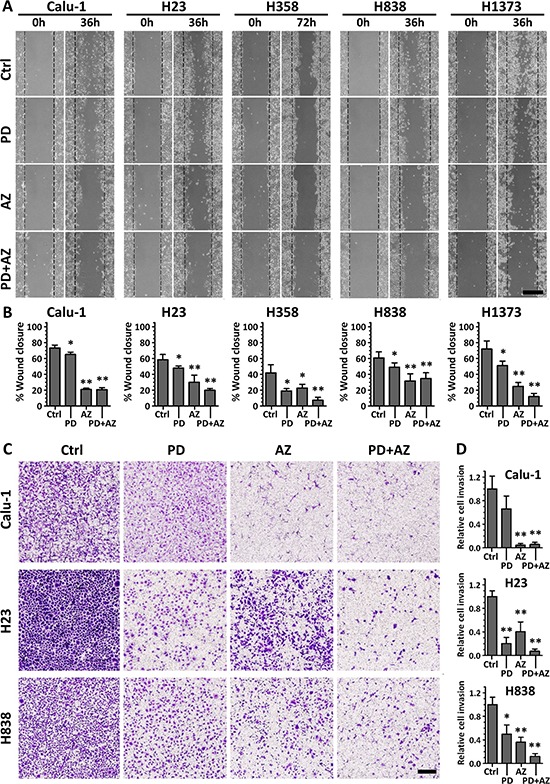
PD0325901 and Saracatinib and the combination treatment reduced cell migratory and invasive activity Cell lines were treated with DMSO control (Ctrl), or with PD (1 μM), AZ (4 μM) or their combination. **A.** for cell migration assays, the wounded areas were imaged at 0 h and 36 or 72 h after the monolayer cultures were scratched. Scale bar: 500 μm. **B.** the gap area of each image was measured, and the wound closure percentages (mean ± SD) were calculated from at least three independent experiments. **C.** for Boyden chamber Matrigel invasion assays, cells that invaded through to the underside of the transwell filter after 48 h incubation under various drug treated conditions were fixed, crystal violet stained and imaged. Scale bar: 250 μm. **D.** cell count analysis was performed for each image and the relative cell invasion (mean ± SD) of each treated group compared to the Ctrl group of each cell line was calculated from at least three independent experiments. *,*P* < 0.05; **,*P* < 0.01 compared with Ctrl group.

We further performed cell migration (scratch) assays and Boyden chamber Matrigel invasion assays on these cell lines to determine whether the MET induction by PD0325901 and Saracatinib could promote a functional response by limiting the cell migratory and invasive behavior. Both PD0325901 and Saracatinib induced MET were able to significantly reduce the cell migratory behavior of the cell lines compared with untreated controls (Fig. 4A; Fig. 4B). In particular, we observed that Saracatinib treatment itself was very effective in reducing cell migration. We attributed this to the fact that Saracatinib potently nullified the phosphorylation of FAK and PXN via disruption of the FAK-SRC-PXN signaling cascade (Fig. 2), and both FAK and PXN played vital roles in cell motility through regulation of focal adhesion dynamics [20]. Interestingly, the combination treatment was observed to be the most effective in further limiting the migratory potential of H23, H358 and H1373 cell lines.

For invasion assay, Matrigel-invasive cell lines Calu-1, H23 and H838 were tested (Fig. 4C; Fig. 4D). We observed that both PD0325901 and Saracatinib induced MET were effective in reducing the cell invasive behavior of these cell lines, despite exhibiting differential anti-invasive sensitivity in response to either of the drug. Nevertheless, the combination treatment was shown to be the most potent in abrogating cell invasion in these cell lines (0.06 ± 0.04 for Calu-1, 0.07 ± 0.04 for H23 and 0.12 ± 0.05 for H838, relative to their respective untreated controls). These functional studies highlighted the complementary inhibitory effects of the PD0325901 / Saracatinib combination treatment.

### PD0325901 and Saracatinib exerted predominantly cytostatic growth arrest in NSCLC lines

**Figure 5:**
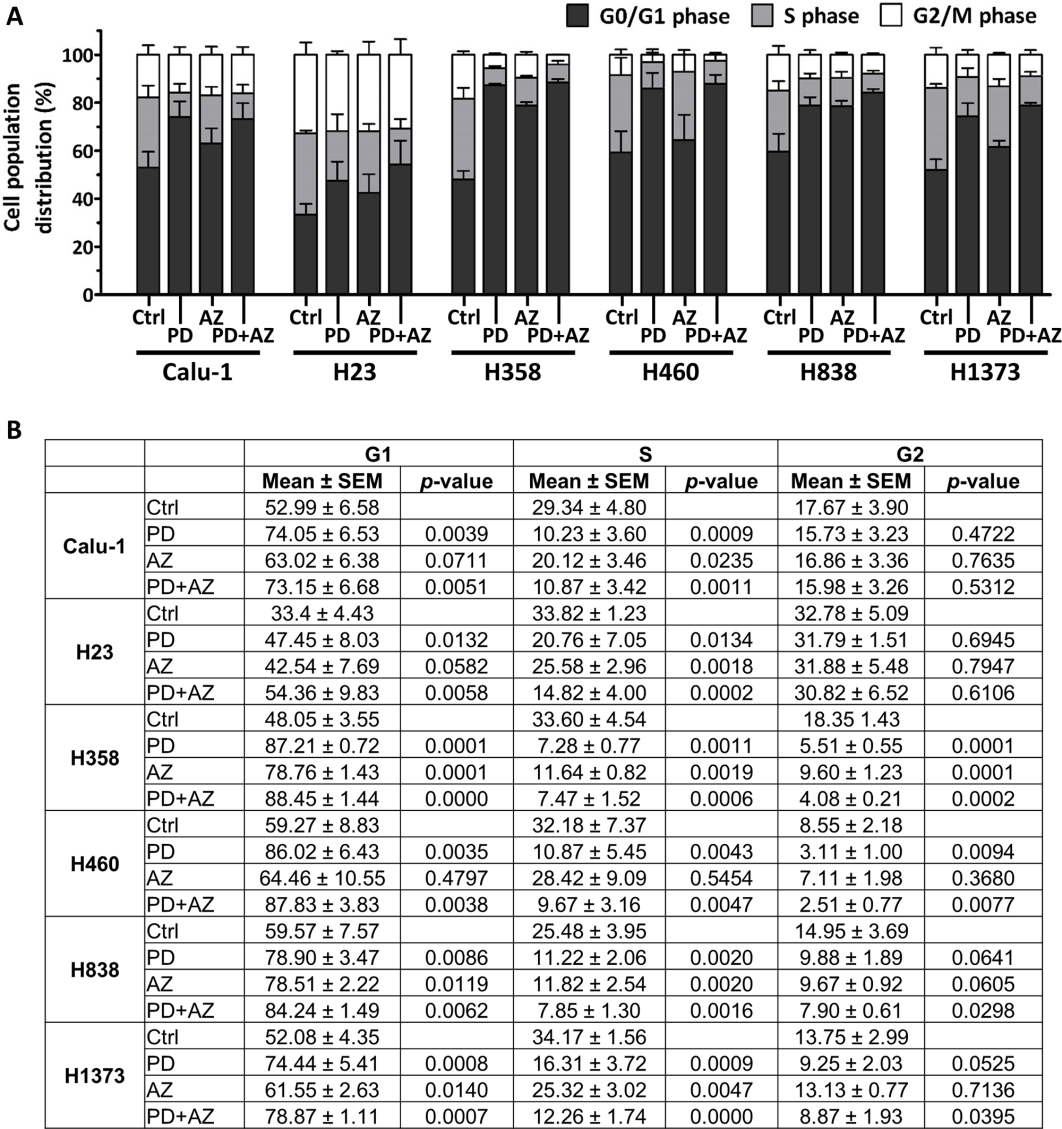
PD0325901 and Saracatinib and the combination treatment primarily exerted G1-phase cell cycle arrest in NSCLC lines Cell cycle analysis was performed on cell lines after treatment with DMSO control (Ctrl), or with PD, AZ or their combination for 48 h. Graph **A.** and table **B.** show the cell cycle distribution (mean ± SD) of each treatment group, calculated from at least three independent experiments. Statistical analyses were performed on treated groups as compared with Ctrl group.

We next investigated on how cell cycle progression was regulated by PD0325901 and Saracatinib in these NSCLC lines. Cell-cycle analysis from Propidium Iodide staining experiments indicated that both PD0325901 and Saracatinib exerted predominantly cytostatic effect and induced significant G1-phase accumulation in the cell lines (Fig. 5A; Fig. 5B). In particular, we observed that PD0325901-treated cells in general exhibited a very strong G1-phase accumulation and S-phase depletion as compared with Saracatinib-treated cells. Interestingly, PD0325901 and Saracatinib combination treatment were observed to maintain or further augment the G1-phase accumulation in the cell lines tested.

### PD0325901 and Saracatinib combination suppressed anchorage-independent colony growth in NSCLC lines

For the cell lines (Calu-1, H23, H460 and H1373) that were able to grow under anchorage-independent conditions in soft agar assays (Supplemental Fig. 3), we observed that Saracatinib treatment alone can only modestly inhibit colony growth, with only Calu-1 responding with IC_50_ < 1 μM. PD0325901 treatment alone, on the other hand, was potent in reducing anchorage-independent colony growth, with all cell lines responding with IC_50_ < 1 μM (Supplemental Table 1). This correlates with the strong cytostatic growth arrest effect exerted by PD0325901 in the NSCLC lines, as shown previously (Fig. 5A). The PD0325901 and Saracatinib combination treatment was also effective in suppressing anchorage-independent colony growth. Interestingly, we analyzed that the combination treatment can substantially reduce the effective IC_50_ of Saracatinib, without increasing the effective IC_50_ of PD0325901 in the combination. The drug combination was also observed to act mostly synergistically on Calu-1, H23 and H460 and additively on H1373.

### PD0325901 and Saracatinib delayed tumor growth and induced E-cadherin expression in xenograft tumors

**Figure 6:**
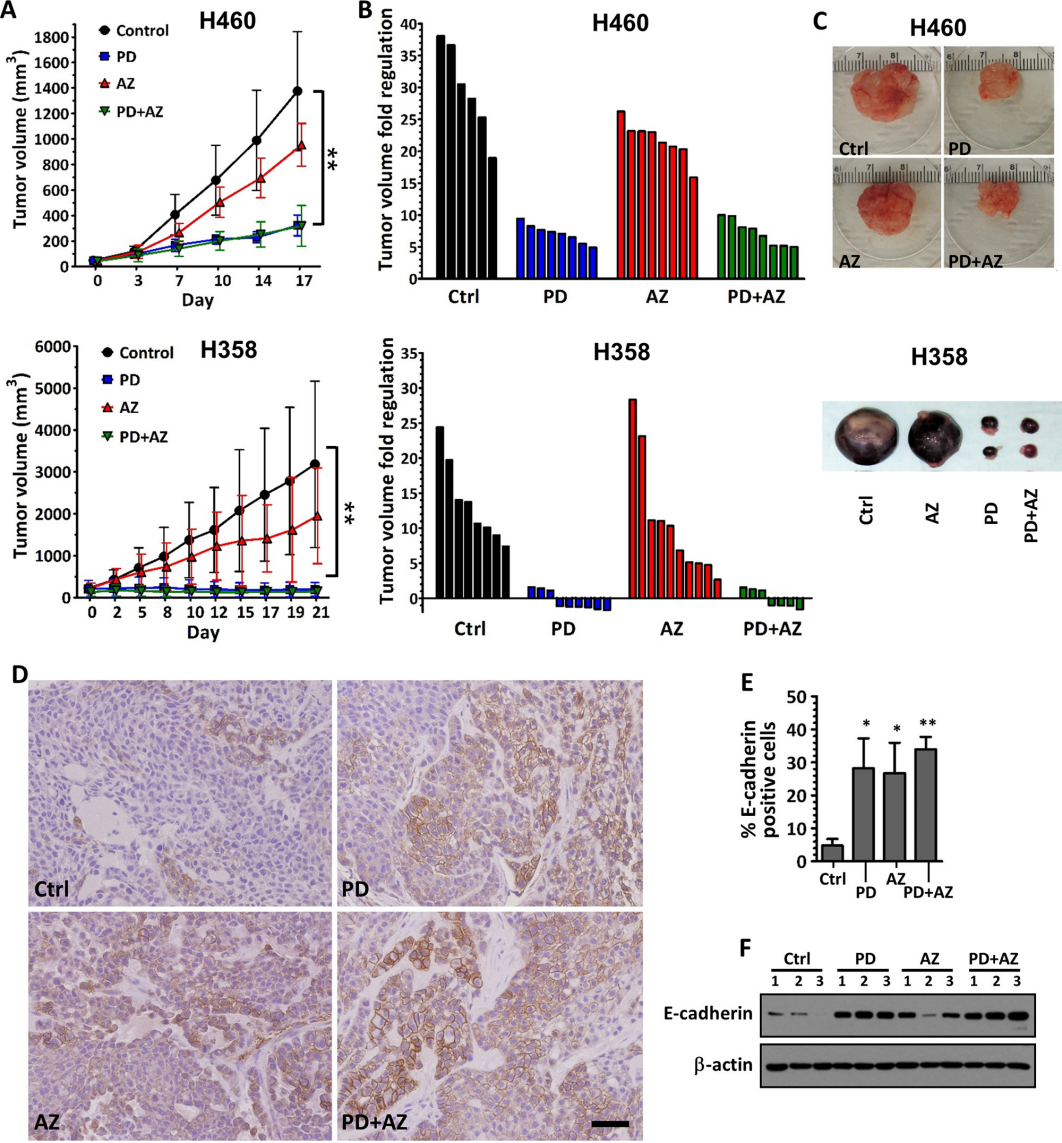
Inhibition of tumor growth and induction of E-cadherin expression in xenograft tumors by PD0325901 or Saracatinib treatment alone or in combination H460 and H358 cells were subcutaneously implanted into the flanks of nude mice. Treatment was started when average tumor volume reached 50 mm^3^ (for H460) or 200 mm^3^ (for H358) in size. Vehicle control only (Ctrl), PD (12.5 mg/kg), AZ (50 mg/kg) or their combination was administered daily. **A.** tumor volumes were measured biweekly and data were expressed as mean ± SD of 6 to 8 tumors per group. *P* < 0.01 compared with Ctrl group. **B.** waterfall plots showing the tumor volume fold regulation (relative to initial tumor volume) for the individual tumors in each treatment group after 17 days treatment. **C.** images of representative H460 and H358 tumors from each treatment group. **D.** E-cadherin expression levels were also assessed in the H460 tumors by IHC. Representative examples were shown. Scale bar: 50 μm. **E.** E-cadherin expression in the IHC images of H460 tumors was analyzed by quantifying the number of E-cadherin-positive cells (brown) compared to the total cells. Bar graph showing the mean percentage of E-cadherin-positive cells ± SD of 3 tumors per group. *,*P* < 0.05; **,*P* < 0.01 compared with Ctrl group. **F.** westernblot showing relative E-cadherin expression of whole tumor lysates for each treatment group of H460 tumor (*n* = 3 per group). β-actin shown as loading control.

We evaluated the single and combinatorial effects of PD0325901 and Saracatinib co-treatment *in vivo* in H460 and H358 xenograft models, using doses previously documented to result in therapeutically-relevant concentrations [21, 22]. Tumors-bearing mice were randomized and administered with vehicle control, PD0325901 or Saracatinib alone, or with PD0325901 / Saracatinib combination. H460 and H358 tumors in the vehicle control group grew rapidly during the course of the experiment, reaching 29.6 ± 7.2 and 13.6 ± 5.8 tumor volume fold change respectively at assay end point. Saracatinib treatment alone resulted in moderate delay in both H460 and H358 tumor growth (21.7 ± 3.0 and 10.8 ± 8.5 tumor volume fold change respectively), while PD0325901 alone or combination treatments resulted in substantial tumor growth inhibition in H460 tumors (7.10 ± 1.5 and 7.20 ± 2.0 tumor volume fold change respectively) and tumor regression in H358 tumors (0.95 ± 0.33 and 1.07 ± 0.29 tumor volume fold change respectively) (Fig. 6A; Fig. 6B; Fig. 6C). Minimal weight loss was observed in the mice in all treatment groups, suggesting that this combination strategy was well-tolerated (data not shown).

We observed through immunohistochemistry staining that E-cadherin expression was significantly induced in the H460 tumors treated with PD0325901 or Saracatinib, compared with vehicle control. Interestingly, the PD0325901 / Saracatinib combination treatment also induced a moderately higher induction of E-cadherin, compared with the single drug treatments (Fig. 6D; Fig. 6E). We further confirmed from the whole tumor lysates that E-cadherin expression was increased in PD0325901, Saracatinib and drug combination treated tumors, compared with vehicle control (Fig. 6F).

## DISCUSSION

The present study shows that SRC and MEK co-inhibition by Saracatinib and PD0325901 respectively can be broadly effective in tumor growth control of a wide panel of NSCLC cell lines. In addition, we show that PD0325901 and Saracatinib are potent EMT modulators and both are effective in inducing a MET response in Snail1 positive NSCLC cell lines. Furthermore, we demonstrate that the co-inhibition of SRC and MEK pathways results in a MET phenotype that is accompanied by a decrease in Snail1 and an increase in E-cadherin expression with low cell migratory ability. These data provide proof-of-concept for the use of PD0325901 and Saracatinib in combination to combat invasive growth in NSCLC.

The activation of the SRC kinase is known to promote the activity of angiogenesis, proliferation, survival and invasion pathways, which leads to the aberrant growth of tumors. The active SRC pathway has also been documented in upwards of 50% of tumors derived from several cancers, including NSCLC [23]. Therefore, this potential target has been extensively investigated in NSCLC, and over the past decade, several clinical studies have evaluated the use of selective SRC inhibitors such as Saracatinib for treating NSCLC [24–27]. However, although Saracatinib was well tolerated in cancer patients and reduction in tumor SRC activity was observed in phase I trial [24], it was later shown to be ineffective as single agent therapy, as objective responses to Saracatinib were not observed in phase II trials in NSCLC [25] as well as other cancers [26, 27]. Our *in vivo* experiments also confirmed that Saracatinib treatment alone only resulted in modest growth delay with no tumor shrinkage (Fig. 6).

Likewise, the MAPK signaling cascade also plays critical roles in the regulation of cell proliferation, survival, differentiation, motility, and angiogenesis. It serves a pivotal role in oncogenesis and growth of transformed cells, and investigations on blocking the MAPK pathway via MEK inhibitors such as PD0325901 have come to the forefront as an exciting approach in cancer therapeutics [28]. However, although PD0325901 demonstrated antitumor activity in preclinical xenograft models [29] and preliminary clinical activities on melanoma and NSCLC patients in phase I trial [30], the phase II study on patients with advanced NSCLC failed to show any objective responses [31]. Due to lack of responses coupled with the safety issues, current studies focus on PD0325901 schedule and the use of rational combination strategies [31, 32]. Our study suggests that SRC and MEK co-inhibition by Saracatinib and PD0325901 can be a proof-of-concept treatment strategy since the combination acts mostly synergistically in inhibiting cell proliferation across a wide panel of NSCLC cell lines (Fig. 1). In addition, we show that PD0325901 can be co-administered at a lower dose ratio compared to Saracatinib, which may circumvent the dose safety issues related to PD0325901.

The role of EMT as one of the important non-genetic mechanisms that drives tumor cell dissemination and drug ineffectiveness to cancer therapy has been intensively researched [12–15]. The extent of EMT across various cancers has recently been reported to vary widely, highlighting the heterogeneity of cancers [33]. We hypothesize that treatments that modulate the EMT status of tumors towards a more defined state (i.e. epithelial state) may reduce the metastatic aggressiveness of the cancer and make the tumors more susceptible to conventional chemotherapy. It is well-accepted that a repertoire of dysregulated signaling pathways is responsible for the induction of EMT in cancers [34–36]. In addition, the development in targeted therapeutics to treat pathways driven cancers have rapidly revolutionized anti-cancer therapies [37, 38]. However, the EMT modulating properties of many clinically tested targeted drugs have not been validated in human cancers. Our previous high-throughput screening study to identify EMT modulators had uncovered several targeted compounds (PD0325901 and Saracatinib among them) that could potently interfere with growth factor induced EMT signaling [14]. In this study, we further validate that both PD0325901 and Saracatinib are potent EMT modulators and individually, each compound can differentially induce a MET response in Snail1 positive NSCLC cell lines. Although the mechanisms driving these differential MET still remains to be elucidated, overall, we show that the PD0325901 and Saracatinib combination can effectively be used in combination to strongly drive cancer cells towards a more epithelial state through up-regulation of E-cadherin across most of the NSCLC lines tested. This is consistent with findings by two other groups whereby inhibiting both SRC and MEK signaling suppressed the invasive growth and cellular survival of tumor cells [39, 40]. Importantly, we verified that high E-cadherin expression is associated with poor migratory activity in NSCLC cells (Supplemental Fig. 4A; 4C). Although there are controversial reports that show a correlation between high E-cadherin expression with aggressive and metastatic growth in breast and ovarian tumors, the differential impact of E-cadherin expression on invasive growth in different organ types needs further investigation [41, 42]. Furthermore, we show that the high E-cadherin expression did not activate other reciprocal pathways like AKT and MAPK signaling pathway (Supplemental Fig. 4B; 4D). This suggests that PD0325901 and Saracatinib combination serves as a favorable therapeutic strategy that induce MET in NSCLC cells yet overcoming the activation of reciprocal pathways prevalent in drug resistance.

In conclusion, we demonstrate through biomarker expression and functional studies that MET can be enhanced in NSCLC lines when PD0325901 and Saracatinib are used in combination. We also demonstrate that PD0325901 synergized with Saracatinib co-treatment to reduce cell proliferation and tumorigenicity in NSCLC lines. These data support further preclinical and clinical development of combining SRC and MAPK pathways inhibition to treat advanced stage NSCLC.

## MATERIALS AND METHODS

### Cell culture

All lung carcinoma cell lines except PC-9 were obtained directly from the American Type Culture Collection (ATCC) repository. The PC-9 cell line was a gift from A. Ali (CSI, Singapore). Cell lines were authenticated by DNA short tandem repeats analysis using GenePrint 10 kit (Promega). The cell lines were maintained in RPMI-1640 (Nacalai Tesque) growth medium supplemented with 10% fetal bovine serum (FBS, HyClone, Thermo Scientific), 2 mM L-glutamine (Gibco, Life Technologies) and 100 U/ml penicillin-streptomycin (Gibco, Life Technologies) at 37°C in a 5% CO_2_ atmosphere.

### Cell proliferation assay and synergism analysis

Saracatinib (AZ) and PD0325901 (PD) were purchased from Selleck Chemicals. Cells were plated into microtiter culture plates (Greiner) and treated with varying concentrations of PD or AZ alone, or drug combinations at fixed PD to AZ ratios. After 72 h, cells were lysed with CellTiter-Glo luminescent cell viability assay reagent (Promega) and luminescence was read using a microplate reader (Infinite M200 Pro, Tecan). Percent cell proliferation was calculated relative to DMSO control treated cells.

Sigmoidal dose-response curve fitting for each treatment condition were analyzed using GraphPad Prism 5 software. Drug concentrations that result in cell proliferation inhibition of between 50% and 80% (i.e. IC_50_ to IC_80_) for each treatment condition were then calculated and tabulated for synergism analysis.

Combination index (CI) for PD and AZ combination was calculated based on the Loewe Additivity equation [18]. Briefly, for a cell growth inhibition effect of E_y_:

$$\text{CI}\left({\text{E}}_{\text{y}}\right)=\frac{{\text{d}}_{\text{PD}}}{{\text{D}}_{\text{PD}}}+\frac{{\text{d}}_{\text{AZ}}}{{\text{D}}_{\text{AZ}}}=\frac{\text{r}\times {\text{d}}_{\text{AZ}}}{{\text{D}}_{\text{PD}}}+\frac{{\text{d}}_{\text{AZ}}}{{\text{D}}_{\text{AZ}}}$$

where d_PD_ and d_AZ_ are the respective combination doses of PD and AZ that will achieve y% cell growth inhibition effect; D_PD_ and D_AZ_ are the corresponding single doses for PD and AZ to result in the same effect; and r is the PD:AZ concentration ratio in the drug combination. The CI values for between 50% to 80% growth inhibition effect, CI(E_50–80_), was tabulated for the drug combination treatment on each cell line.

### Western blots

E-cadherin antibody was purchased from BD Biosciences. Plakoglobin and Paxillin (PXN) antibodies were purchased from Millipore. Antibodies against Snail1, mitogen-activated protein kinase (ERK1/2), phospho-p44/42 ERK1/2, SRC, phospho-SRC (pY416), focal adhesion kinase (FAK), phospho-PXN (pY118), phospho-Akt (pS473), β-actin and HRP-conjugated secondary antibodies were purchased from Cell Signaling Technology. Phospho-FAK (pY861) antibody was purchased from Thermo Scientific.

Cells are grown on 6-well plates and treated with compounds or DMSO as control for 48 h. Cells were rinsed with PBS and lysed with RIPA buffer supplemented with protease / phosphatase inhibitor cocktail (Promega). Protein concentration was quantified and equalized protein loads were resolved with Bio-Rad SDS-PAGE system using 8% to 12% polyacrylamide gels and transferred to PVDF membranes. Immunoblotting were performed with the antibodies listed above and bound antibodies were detected by chemiluminescence using Amersham ECL Prime Western Blotting reagent (GE Healthcare).

### Immunofluorescence imaging

Cells were plated into black, clear bottom 96-well microtiter plates (Greiner). When the cells reach about 50% confluency, the cultures were then treated with compounds or DMSO as control. After 48 h treatment, cells were gently rinsed with PBS, fixed with 4% paraformaldehyde in PBS for 15 min and permeabilized with ice-cold methanol for 10 min. Samples were then blocked with blocking buffer (2% FBS, 0.3% Triton-X in PBS) for 30 min, followed by immunostaining with E-cadherin (1:500) and Plakoglobin (1:500) primary antibodies in blocking buffer overnight at 4°C. Samples were then incubated with Alexa Fluor 488 secondary antibodies (1:1000, Molecular Probes, Life Technologies) for 2 h at 25°C, followed by Hoechst 33342 nuclear staining (1:2000, Sigma) for 15 min. The wells were then washed and kept in PBS. The plates were sealed with aluminum sealing films (Axygen, Fisher Scientific) and stored at 4°C prior to fluorescence imaging.

Plates were imaged using a confocal microplate imager (MetaXpress Ultra, Molecular Devices) with 40× objective, 405 nm laser / 417–477 nm filter cube configuration for nuclei imaging, and 488 nm laser / 525/50 nm filter cube configuration for E-cadherin and Plakoglobin imaging. At least 8 sites were imaged for each well. Image sets were analyzed with ImageJ software using an image processing workflow described previously [14, 43]. Object segmentation was performed and the E-cadherin or Plakoglobin fluorescence intensity of each cell was quantified. Fluorescence intensity of compound treated cells was calculated relative to DMSO control treated cells.

### Cell migration assay

Cells were plated into 6-well plates and grown to confluency. A 200 μl pipette tip was used to make linear scratches on the cell monolayer. The wells were then gently washed with media to remove unattached cells and refreshed with culture media containing test compounds or DMSO control. Suitable areas centered on scratch regions with appropriately 0.60 – 0.75 mm wound gap, were selected for wound closure observation. Images of selected areas were acquired at 0 h (T1) and at the end of the cell migration experiment (T2: 36 h for Calu-1, H23, H838 and H1373; and 72 h for H358), using an optical imaging microscope at 4× magnification (Olympus CKX41/C-5060). The wound gap area for each image was measured using ImageJ software. For each T1 and T2 image set, the wound closure percentage was calculated using the formula: (Area_T1_ − Area_T2_) / Area_T1_ × 100%.

### Cell invasion assay

Cell invasion assay was performed using 8 μm pore, 24-well format transwell inserts (Corning). Transwell membranes were coated with 0.1 ml of 0.25 mg/ml matrigel (BD Biosciences) in PBS and incubated overnight at 37°C. The following day, 40 × 10^3^ cells in 1% FBS culture media were seeded into each transwell insert, while 0.5 ml of 10% FBS culture media containing test compounds or DMSO control was added into the lower chambers. Following a 48 h incubation to allow cell penetration, non-invaded cells in the transwell insert were removed using cotton swab and the invaded cells on the membrane underside were fixed with methanol and stained with 0.1% crystal violet in PBS and air dried. For each membrane, five non-overlapping brightfield images were acquired with an optical imaging microscope at 4× magnification (Olympus IX71/DP71). The number of cells in each image was then counted using ImageJ software. Percent cell invasion was calculated relative to DMSO control treated cells.

### Cell cycle analysis

Cell cycle fractions were determined through propidium iodide (PI) nuclear staining. Briefly, cells are grown on 6-well plates and treated with compounds or DMSO as control for 48 h. Cells were then harvested, fixed with 70% cold ethanol, and stained with PI staining buffer (0.03 mg/ml PI, 0.1 mg/ml RNAse A, 0.1% Triton-X in PBS) for 30 min at 25°C. Flow cytometry was performed using BD LSR II flow cytometer (BD Biosciences). At least 10,000 cell events were collected per sample. Cell cycle analysis was performed using FlowJo software (Tree Star).

### Anchorage independent assay

0.5 ml of cell suspension in 0.36% agarose containing culture media was plated into each well on the top of existing 0.6% bottom agarose in 24-well tissue culture plates. 0.5 ml of culture media containing compound or compound combinations at various doses was then loaded into each well. Plates are incubated in a 37°C, 5% CO_2_ incubator for a period of 2 – 4 weeks to allow cell colonies to grow large enough to be visualized through MTT tetrazolium dye staining. At the assay endpoint, 0.04 ml of 5 mg/ml MTT solution (Sigma) were added into each well and further incubated at 37°C for 3 h. Cell colony images of each well were acquired using a flatbed scanner (Epson). The number of colonies formed in each well was then quantified using ImageJ software. Percent cell colony formation was calculated relative to DMSO control treated cells.

### Tumor xenograft

All animal work adhered to the Institutional Animal Care and Use Committee (IACUC) guidelines on animal use and handling. Female athymic BALB/c nude mice between 6 to 8 weeks old were maintained and handled in a pathogen-free environment under controlled conditions and received food and water ad libitum. Subconfluent H460 or H358 cells were resuspended in PBS at 40 × 10^6^ cells/ml, and 0.1 ml cell suspension was injected subcutaneously into the flanks of each animal. The tumors were measured with vernier calipers twice a week and the volume was calculated using the modified ellipsoidal formula (length × width^2^)/2. Median tumor size at initiation of drug treatment was 50 mm^3^ (for H460 cells) and 200 mm^3^ (for H358 cells). Both PD and AZ were prepared in 1% polysorbate 80 (Sigma). The mice were randomized into four groups of five mice and gavage fed with vehicle control, or 12.5 mg/kg PD, or 50 mg/kg AZ, or 12.5 mg/kg PD and 50 mg/kg AZ combination. All drugs were administered once a day, 5 days a week. The mice were euthanized and the xenograft tumors harvested after 3 weeks of drug treatment.

### Immunohistochemistry

Xenograft tumors from control and compounds administered mice were fixed in formalin immediately after surgical excision, and subsequently paraffin embedded and sectioned. Tumor sections were deparaffinized using standard histologic procedures and stained with E-cadherin antibodies. Color development was performed using EnVision+ System-HRP (DAB) kit (Dako, Agilent Technologies) according the manufacturer's recommendation. Cell nuclei were counterstained with Hematoxylin (Thermo Scientific). Slide images were acquired with an optical imaging microscope at 20× magnification (Olympus IX71/DP71). Images were acquired from 3 tumor samples each for every treatment group.

Images were analyzed with ImageJ software using an image processing workflow described previously [43, 44]. Briefly, Hematoxylin and DAB stain color-separated images were derived from the original image using ImageJ Color Deconvolution plugin. Object segmentation using the Particle Analyzer plugin was then performed to identify the individual cellular regions in the image and the DAB intensity of each cell region in the image was then tabulated. E-cadherin staining was analyzed as the percentage of E-cadherin positive cells in each slide image. At least 20 images were analyzed for each tumor sample.

### Transfections

For gene knockdown, CDH1 siRNA (sequence: 5′-GGCCUGAAGUGACUCGUAATT-3′) and AllStar negative control siRNA were obtained from Qiagen. siRNA transfection was conducted with JetPRIME reagent (Polyplus Transfection). For gene overexpression, CDH1-GFP and p-CMV-entry empty vector plasmid constructs were obtained from OriGene Technologies. Plasmid transfection was conducted with ViaFect reagent (Promega).

### Statistical analysis

All data were presented as mean ± standard deviation (SD). The statistical significance of the data obtained was analyzed by two-way ANOVA for tumor xenograft results and Student's *t*-test for all other results. All statistical tests were 2-sided and the significance level was set at *P* < 0.05.

## SUPPLEMENTARY FIGURES, TABLE AND LEGENDS


