# Supplementary material for: Combinatorial treatment using targeted MEK and SRC inhibitors synergistically abrogates tumor cell growth and induces mesenchymal-epithelial transition in non-small-cell lung carcinoma
Source: Oncotarget. 2015 Aug 17;6(30):29991–30005. doi: 10.18632/oncotarget.5031 (PMC4745777; doi:10.18632/oncotarget.5031)
Supplement: Supplementary file 1 [file oncotarget-06-29991-s001.pdf]

SUPPLEMENTARY FIGURES, TABLE AND LEGENDS

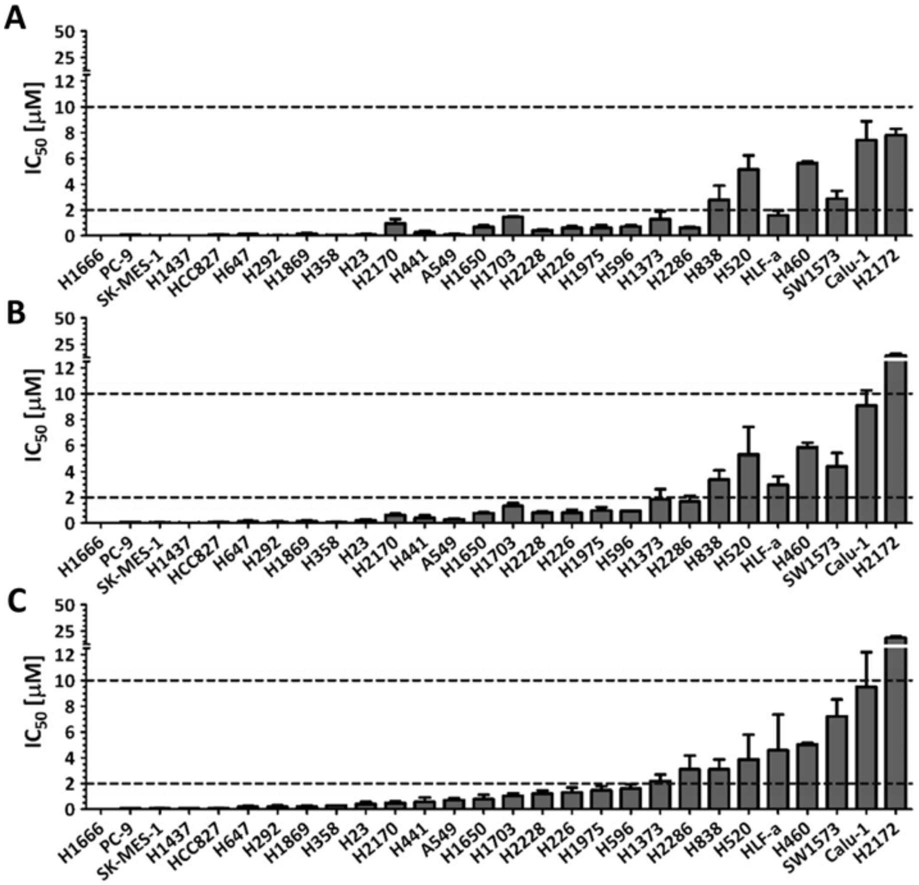

Supplementary Figure S1: Cell proliferation IC_{50} plots (mean ± SD) for a panel of 28 NSCLC cell lines treated at fixed PD0325901 to Saracatinib ratios of 4:1 A. 1:1 B. and 0.25:1 C. for 72 h. Data were tabulated from three independent experiment sets.

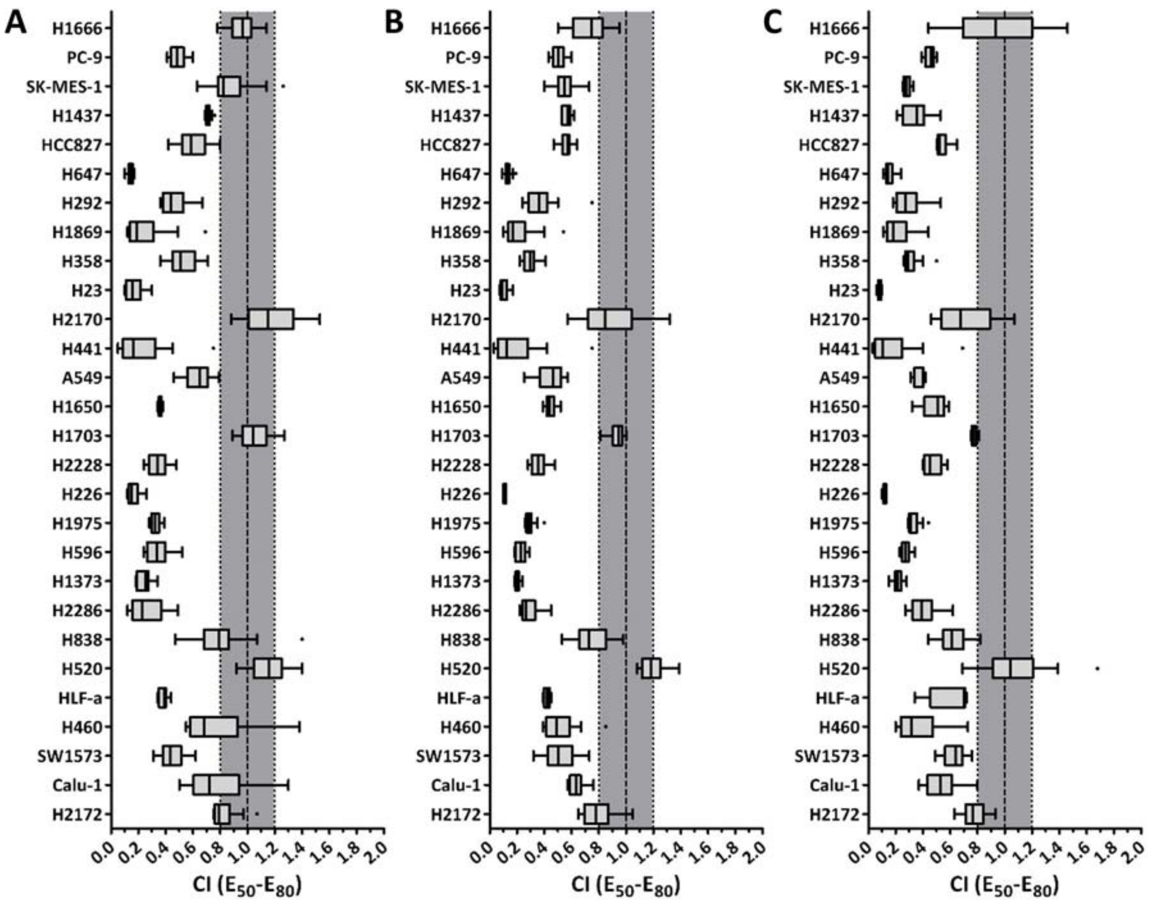

Supplementary Figure S2: Combination index (CI) box plots of PD0325901 and Saracatinib co-treatment at the ratios of 4:1 A. 1:1 B. and 0.25:1 C. on the panel of 28 NSCLC cell lines. Data were tabulated from three independent experiment sets. Combination index of CI < 0.8 indicates synergism, CI from 0.8 to 1.2 indicates additive effect, and CI = 1.2 indicates antagonism.

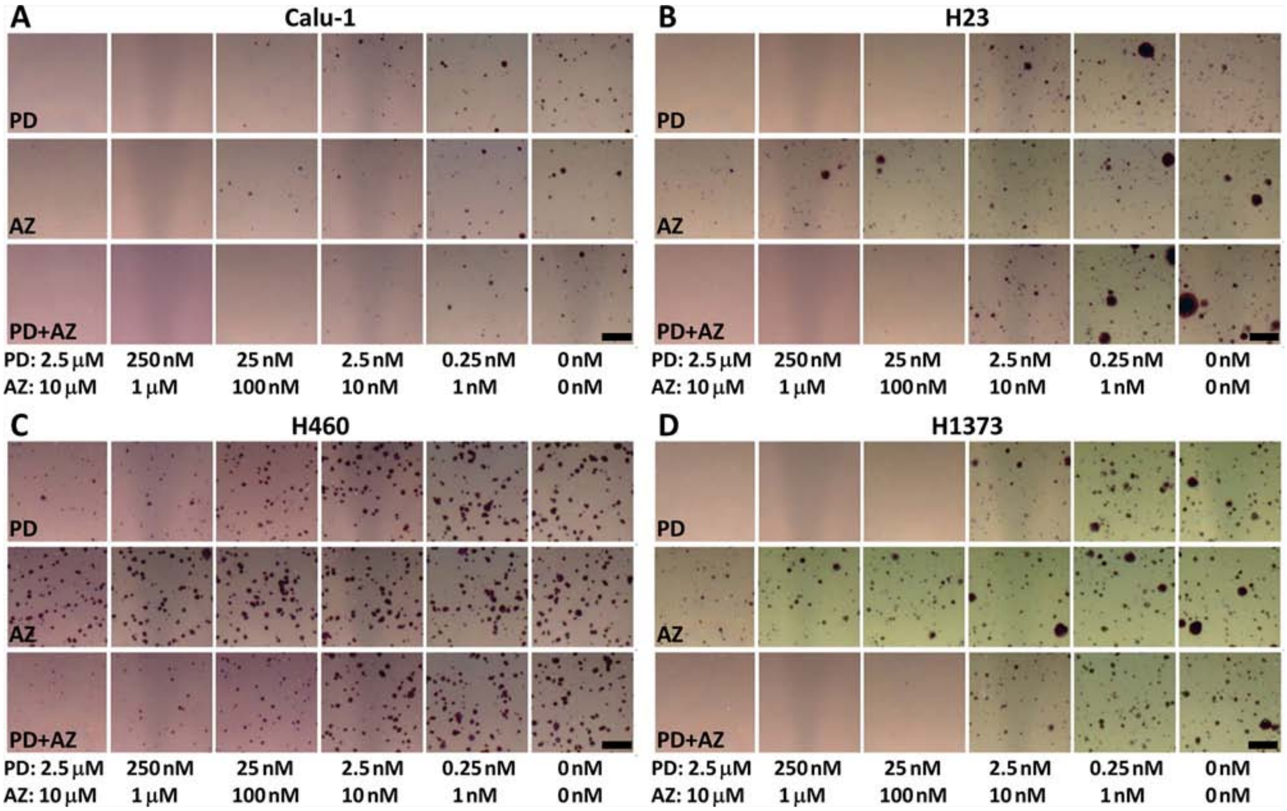

Supplementary Figure S3: Suppression of anchorage-independent growth by PD0325901 and Saracatinib combination treatment. NSCLC lines were grown in soft agar in the presence of various concentrations of PD, AZ or their combination at a fixed ratio of 0.25:1. Representative images shown highlighted the dose-dependent colony inhibitory effect of the various treatments on Calu-1 **A.** H23 **B.** H460 **C.** and H1373 **D.** Scale bars: 1 mm.

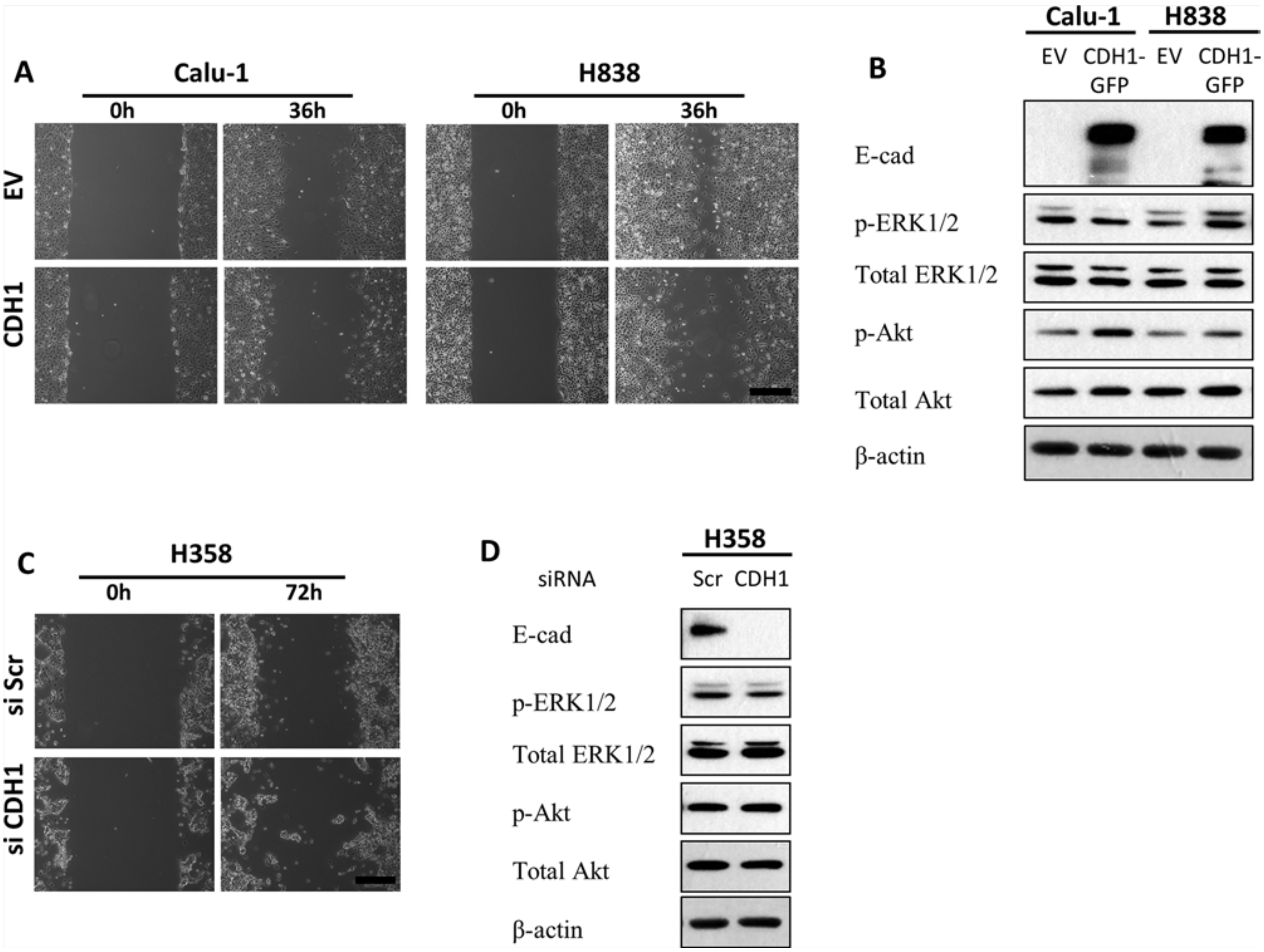

Supplementary Figure S2: E-cadherin expression reduced migratory activity and did not promote tumor progression in NSCLC cells. Calu-1 and H838 cells were transfected with CDH1-GFP or pCMV-entry plasmids (1 ug plasmid in each reaction) **A.** and **B.** H358 cells were transfected with CDH1 or negative control siRNA (50 nM of siRNA in each reaction) **C.** and **D.** Cell migration assays were conducted 24 h after transfections. The wounded areas were imaged at 0 h and 36 h (A, for Calu-1 and H838) or 72 h (B, for H358) after the monolayer cultures were scratched. Representative images were shown. Scale bar: 500 μm. Westernblot was performed to validate the efficiency of overexpression (B) and siRNA knockdown (D). Effects of the transfection on phospho-ERK1/2 and phospho-Akt were also shown. (n = 3 per group). β-actin shown as loading control.

Supplementary Table S1: Anchorage-independent cell colony formation IC_{50} data of the cell lines treated with PD, AZ or PD+AZ combination

Cell line	Single compound treatment		PD + AZ combination treatment		PD + AZ combination index range
	PD IC_{50} (μM)	AZ IC_{50} (μM)	PD IC_{50} (μM)	AZ IC_{50} (μM)	
Calu-1	0.0053 ± 0.0016	0.038 ± 0.016	0.0024 ± 0.0009	0.0095 ± 0.0035	0.48 – 0.71
H23	0.0066 ± 0.0059	4.6 ± 2.1	0.0060 ± 0.0009	0.024 ± 0.004	0.59 – 1.08
H460	0.33 ± 0.04	29.8 ± 2.5	0.19 ± 0.05	0.75 ± 0.18	0.35 – 0.78
H1373	0.0037 ± 0.0002	13.5 ± 0.4	0.0037 ± 0.0001	0.015 ± 0.001	1.01 – 1.07

Note: NSCLC lines were grown in soft agar in the presence of various concentrations of PD, AZ or their combination at a fixed ratio of 0.25:1 for a period of 2–4 weeks. IC_{50} data (mean ± SD) were tabulated from at least two independent experiment sets. The combination index range for between 50% to 80% colony formation inhibition effect was tabulated for the drug combination treatment on each cell line. Combination index of CI < 0.8 indicates synergism, CI from 0.8 to 1.2 indicates additive effect, and CI > 1.2 indicates antagonism.
